# Estimating Recent Local Impacts of Sea-Level Rise on Current Real-Estate Losses: A Housing Market Case Study in Miami-Dade, Florida

**DOI:** 10.1007/s11113-018-9473-5

**Published:** 2018-06-26

**Authors:** Steven A. McAlpine, Jeremy R. Porter

**Affiliations:** 1 Quantitative Methods in the Social Sciences, Columbia University, New York, NY USA; 2 Environmental Health Sciences, Columbia University’s Mailman School of Public Health, New York, NY USA; 3 City University of New York’s Brooklyn College and Graduate Center, CUNY Institute of Demographic Research, New York, NY USA

**Keywords:** Sea-level rise, Property value, Climate change, Community impacts, Disaster

## Abstract

Sea-Level Rise (SLR) Projections from the National Oceanic and Atmospheric Administration (NOAA) and the U.S. Army Corp of Engineers (USACE) indicate increasing, and imminent, risk to coastal communities from tidal flooding and hurricane storm surge. Building on recent research related to the potential demographic impacts of such changes (Hauer et al. 2016, in Nat Clim Chang 3:802–806, 2017; Neumann et al. 2015; Curtis and Schneider in Popul Environ 33:28–54, 2011), localized flooding projections in the Miami Beach area (Wdowinski et al. in Ocean Coast Manag 126:1–8, 2016) and projected economic losses associated with this rise in projected SLR (Fu et al. Ocean Coast Manag 133:11–17, 2016); this research investigates the accrued current cost, in terms of real-estate dollars lost, due to recurrent tidal flooding and projected increases of flooding in Miami-Dade County. Most directly related to this line of research, Keenan et al. (2018) have recently produced results indicating that Climate Gentrification is taking place in Miami, FL with higher elevations in flood prone areas appreciating at a higher rate. In that vein of thinking, we seek to answer a question posed by such research: *What is the actual accrued loss to sea*-*level rise over the recent past*? To answer this question, we replicate well-documented estimation methods by combining publicly available sea-level rise projections, tide gauge trends, and property lot elevation data to identify areas regularly at risk of flooding. Combining recent patterns of flooding inundation with future forecasts, we find that properties projected to be inundated with tidal flooding in 2032 have lost $3.08 each year on each square foot of living area, and properties near roads that will be inundated with tidal flooding in 2032 have lost $3.71 each year on each square foot of living area. These effects total over $465 million in lost real-estate market value between 2005 and 2016 in the Miami-Dade area.

## Introduction

Coastal communities represent some of the most valuable property in the U.S. (Fu et al. 2016) and are disproportionately populated when compared to more inland areas of the country (NOAA 2013). Recent research estimates that as many as 16 million people in coastal counties of the U.S. could be affected by the potential 1.8 meters of Sea-Level Rise (SLR) by the year 2100 (Hauer et al. 2016). As part of that process, SLR is becoming recognized as a push-factor in migration and is likely to play a role in significantly reshaping the distribution of population in the country away from immediately coastal areas (Hauer 2017). Globally, estimates of populations at risk are as high as 315–411 million people in low elevation coastal regions under varying assumptions of population growth by the year 2060 (Neumann et al. 2015). Consequently, there is a growing body of research in this area, although there is a considerable debate among researchers around the arrival, timeframe, and magnitude of SLR consequences. That debate centers on issues such as the manner in which populations will respond to changing coastlines and variations in risk to tidal/storm inundation (Keenan et al. 2018; Hauer 2017), what local and national governments might do to adapt to rising seas (Hinkel et al. 2014; Swiss 2013; Nicholls and Cazenave 2010), and what impact mitigation might have on the processes already contributing to global SLR (Strauss et al. 2015). Despite these variabilities, there is consensus that rising seas will create challenges for coastal populations in the relatively near future.

In light of these developing environmental trends, it is important to consider that the communities that exist on the coast are currently home to a disproportionate percentage of jobs, GDP production, and economic trade points of entry/exit (Fu et al. 2016; Kildow et al. 2014), and NOAA’s “Coastal Population Report […] 1970–2020” indicates that there are no signs that this trend of coastal dominance is likely to break anytime soon (NOAA 2013). However, recent research reminds us that while these communities are some of the most prosperous in the country, they are also some of the most vulnerable (Wdowinski et al. 2016). In particular, what makes them so attractive in the first place, their proximity to water, is directly related to their vulnerability to flooding-related natural disasters. As a response, Keenan et al. (2018) have identified patterns of settlement and investment in the Miami-Dade area that signal a sort of “Climate Gentrification” through the redistribution of population and investment into areas within neighborhoods that are less at risk of flooding due to higher elevation. Particularly in neighborhoods near the coast, properties at higher elevations seem to be appreciating at a faster rate than lower elevation counterparts. Hauer et al. (2016) highlighted this type of potential population redistribution at a national scale with a focus on population projections up to the year 2100. In both cases, the movement of populations away from the increasingly vulnerable coast to areas of higher elevation, or lower risk, signals a larger trend associated with retreat. This trend creates shifts in housing market demand, which further necessitates a change in the practical behaviors associated with buying and selling of homes, including the hardship of owning a home that eventually will not sell, or will only sell below the original cost of the home. For these homeowners, the decision to retreat to less risky areas is clouded by the fact that a significant amount of their wealth is tied up in a home that is likely to be depreciating and unable to attract a suitable buyer. Given that research has provided evidence of this phenomenon, the question of the degree to which SLR may have already impacted the home values in coastal communities in Miami-Dade County is investigated here.

Our research builds on a growing body of literature which suggests that SLR is occurring at a more rapid pace than even some of the more liberal projections can account for (see USACE SLR projections for instance) and that coastal communities, and their disproportionately large populations, are already beginning to see the effects (Curtis and Schneider 2011; Hauer 2017). Some experts in this area have provided evidence that we have already hit a ‘tipping-point’ as far back as the mid-2000s and continued increases in SLR are inevitable (Lindsay and Zhang 2005). Consequently, the costs to cities for adaptation to rising seas are likely to grow from an estimated $6 billion in 2005 to $52 billion by 2050 (Hallegatte et al. 2013). On a large scale, these costs are well documented with numerous recommendations for dealing with SLR through processes of mitigation and adaptation (Bierbaum et al. 2014; Lickley et al. 2014; IPCC 2014) with a secondary component focusing on the argument of “protection versus retreat” (Fankhauser 1995). The National Climate Assessment Reports (Bierbaum et al. 2014; Lickley et al. 2014) point out that since discussions around the concrete application of mitigation and adaptation approaches are relatively new in the policy world, few measures have been actually implemented, leading to a lack of evaluations of their potential utility. Importantly though, the discussion has shifted to a place where we are no longer asking the question “Is Climate Changing?” but instead are asking the question “Will Society be able to deal with the Changing Climate?” In addressing those questions, we must also understand the large range of consequences associated with continually rising seas for the foreseeable future.

That being said, few analyses have been conducted at the property level to better understand the parcel-level impact of SLR on individual housing market outcomes (see Keenan et al. 2018 for a recent exception) and those that have looked at these impacts have generally been interested in the impact that permanent land loss due to SLR would have on local housing markets well into the future (Fu et al. 2016). Our current research takes a different approach in that we make use of historical property transactions in Miami-Dade County from 2005–2016 to estimate the actual lost dollars per square foot over that time period, controlling for macroeconomic temporal trends (i.e., the recession around 2008), house characteristics, and community amenities. Ultimately, our models indicate that both current and near-term (15 years out) forecasted flood inundation levels have independently, and negatively, impacted the value of homes in the Miami-Dade County area from 2005 to 2016 when compared to changes in home values for properties that are not at risk of being affected by flooding nor likely to become affected in the near future. These findings were consistent for both flooding levels within the boundaries of property lots and in relation to the flooding levels of roads in the immediate neighborhood vicinity of each property lot.

## Literature Review

Tidal flooding from SLR and its relationship to property devaluation has a potentially interesting, and unique, relationship in that most natural disasters categorized as “environmental risk” (earthquakes, tornadoes, tsunamis, etc.) are rare and relatively unpredictable. However, SLR and the increased property flooding that has occurred as a result are happening at a rate that is both more predictably quantifiable and increasing with some degree of certainty. These trends have drawn attention to the potential costs associated with SLR, storms, and tidal flooding (Kulp and Strauss 2017; Nicholls 2011; Frazier et al. 2010; Shepard et al. 2012; Tebaldi et al. 2012); and the potential threat of shortening the lifespan of some coastal properties (Seidel et al. 2013). The process of SLR is long and gradual and has been occurring since the end of the last ice age due to glacial melt and thermal expansion, both part of natural climate cycles. However, within the last few decades the pace of SLR has accelerated due to temperature trends and associated climate change (Kulp and Strauss 2017; Butler et al. 2016). Few places are the effects of accelerating SLR more evident than in South Florida, where NOAA’s tide gauge at Virginia Key indicates that SLR has increased from a rate of 3 ± 2 mm/year in the decade before 2006, to 9 ± 4 mm/year since (Wdowinski et al. 2016). Wdowinski et al. (2016) research further makes the point that flooding frequency in the city of Miami Beach has risen during that time due to an interaction of tidal, rain, and storm forces and that the media coverage surrounding such events has increased disproportionately as well. The measurable increase in frequency and coverage of such events is seen as directly contributing to other claims that early signals of climate gentrification are taking place in the region (Keenan et al. 2018).

To combat the effects of SLR, localities in South Florida have built pumps and elevated roads (Butler et al. 2016). The costs of such projects are often justified by estimating future property values and the overall economic return, in taxes and dollars spent, of places preserved by the adaptation measures (Fu et al. 2016). The estimates of property values forecasted to be impacted are typically conducted by determining the aggregate amount of property value that will be permanently inundated after 1, 2, 3, or more feet of sea-level rise. We take a different approach to understanding the impact of SLR on property values by instead examining the amount of value that has been historically lost to recent, and near-term, flooding from SLR. The mechanisms through which we expect these shifts to be taking place primarily revolve around property buyers’ concerns about known and expected flooding in the area and the increased attention the phenomenon is getting in the city over the past decade. In other words, in locations where frequently flooding is currently known to be taking place or locations where it is likely to move from infrequent to frequent in the near future, are property values growing at a slower rate than flood non-affected areas?

Methods to disentangle the negative impact of flooding risk from the positive impact of beach proximity when estimating property values have been utilized successfully by a number of researchers, but most directly related to our current research are the hedonic models developed by Bin et al. (2008) which estimated future losses in North Carolina based on SLR scenarios at the time. The research by Bin et al. (2008) is illuminating in its estimation of future economic implications revolving around real-estate losses, but the primary utility of the research for our work is that it was estimated at a property level. This is significantly different than most research in the area which estimating the economic cost of inundation from flooding on a national or global scale (see Neumann et al. 2014; Yohe et al. 1995, 1996, 1999; Darwin and Tol 2001). The large-scale focus of the economic impact of rising seas is in line with the more general examination of these economic impacts of rising seas (see Fu et al. 2016; Hallegatte et al. 2011; Bin et al. 2008, 2011; Parsons and Powell 2001; Michael 2007).

Building on the general economic focus of these past approaches, our research looks specifically at Miami-Dade County, FL. In this context, there is reason to believe that buyers in this region may be increasingly considering elevation and flood risk when purchasing homes (Keenan et al. 2018). Past research on this topic has also been conducted specifically in Broward County (one county north of our study region) and found that many residents are highly aware of the potential risks to their own property due to flooding from current and future SLR (Bolter 2014). Despite the increase in evidence of local awareness, a gap exists between the empirical outcomes associated with perceived risk and actual risk. We draw directly on the public’s awareness of the importance of elevation, distance to shorelines, and flood risk to estimate the impact of such considerations on recent property transaction outcomes.

## Methods

### Purpose

The current research looks to build on the work presented in the previous sections by modeling the historical effect SLR and flooding inundation has had on relative property values in Miami-Dade, FL. Research indicates that there are signals of climate gentrification and correlations between price appreciation and elevation in the region (Keenan et al. 2018) which are directly related to rising sea levels and increased media attention to flooding events (Wdowinski et al. 2016). Furthermore, the literature indicates sea-level rise, and the resulting permanent land loss, is associated with a loss of real-estate value when forecasted into the future (Fu et al. 2016; Bin et al. 2011). Tying these lines of research together, we model the impact on property values of currently measurable and forecasted flooding due to sea-level rise combined with tidal forces by controlling for factors commonly found to be related to home values. We chose transactions occurring from 2005 to 2016 in Miami-Dade County due to the measurable and sudden increase in flooding events, and the likely impact the associated increase in media coverage would have on property transactions.

### Storm Surge from Hurricanes

**Fig. 1:**
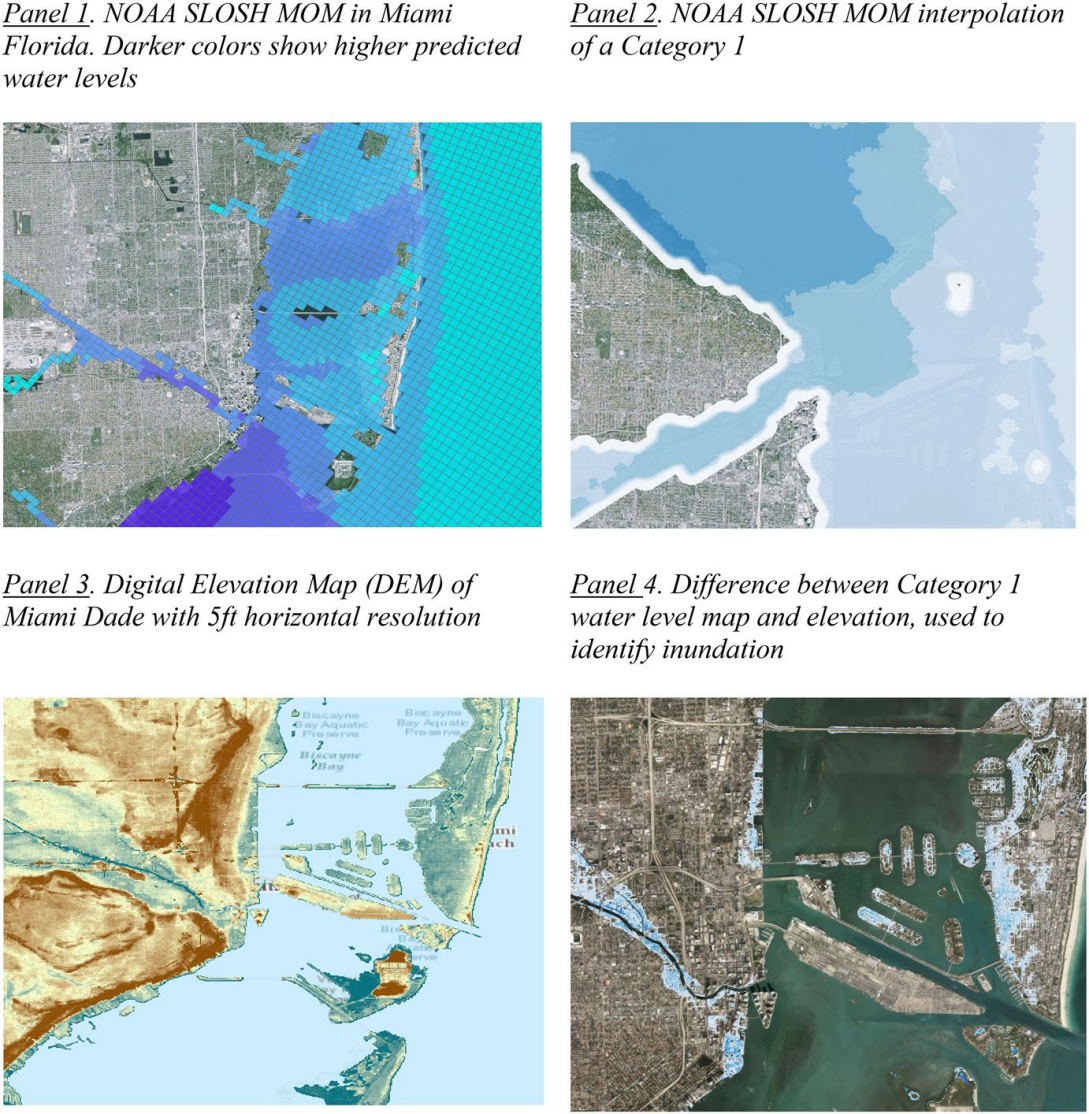
Development of hurricane inundation variable

The literature indicates that properties in Miami-Dade are at risk of flooding from three main sources: storm surge from hurricanes, tidal flooding from sea-level rise and astronomical forces, and flooding due to excess rainwater combined with poor drainage. The first of these sources, storm surge, is a particularly destructive aspect of hurricanes since surge brings water deep onshore by suddenly raising the sea level above coastal barriers such as sand dunes or sea walls. In order to assess the potential impact from storm surge to coastal regions, the National Weather Service (NWS) of NOAA runs computer simulations called Sea Lake and Overland Surges from Hurricanes (SLOSH) models to determine the extent to which hurricanes may produce surge which forces water inland. NOAA makes the outputs of these simulations readily available for the regions, called SLOSH basins, in which the SLOSH models are run. For most basins, the maximum depth of water and elevation of water are available for each category of storm simulated at normal and high tide conditions. SLOSH grids are moderately coarse, meaning they provide accurate average inundation predictions for areas that cover multiple city blocks, but do not show the varying inundation within the block due to variations in elevations (Fig. 1, Panel 1).

To better approximate the local inundation variations within each SLOSH cell, as well as to smooth the transition from one cell to another, an interpolated surface based on the SLOSH grids and a high density of random points were used to sample the SLOSH grid values. The random points were created to oversample each of the spatial locations within each cell to ensure that the entire SLOSH basin could be sampled at an extremely high density. Based on the high density of points with sampled values from the SLOSH grid cells, we were then able to interpolate a high-resolution raster which represented a more accurate presentation of the settlement of water (Fig. 1, Panel 2). That interpolated surface was then differenced from the high-resolution Light Detection and Ranging (LIDAR) Digital Elevation Model (DEM) (at a resolution of 5-foot horizontal) (Fig. 1, Panel 3) to determine areas at risk of inundation by identifying any areas where the SLOSH storm surge in feet, from a Category 1 and Category 3 hurricane was higher than the measured elevation of the DEM. The resolution is necessary given we are interested in maintaining the exact location of water (to the extent permitted by the resolution of the DEM) for the purpose of measuring the most realistic proportion of the lot inundated by each of the flooding scenarios. This approach served two purposes: (1) to create a measure of lot proportion inundated that included more variation than a binary flooding versus not flooding indicator and (2) to increase the reliability of the lot proportion flooded measure by reducing any error that could be potentially introduced by rules associated with assignment of wet versus dry cells due to a lower resolution. Ultimately, the SLOSH interpolated surface was then differenced against the DEM and flooding inundation levels were determined for areas where the SLOSH estimation was above the DEM (Fig. 1, Panel 4). This storm surge inundation estimation was estimated for both C1 and C3 hurricane scenarios.

### Flooding from Tidal Events

**Fig. 2:**
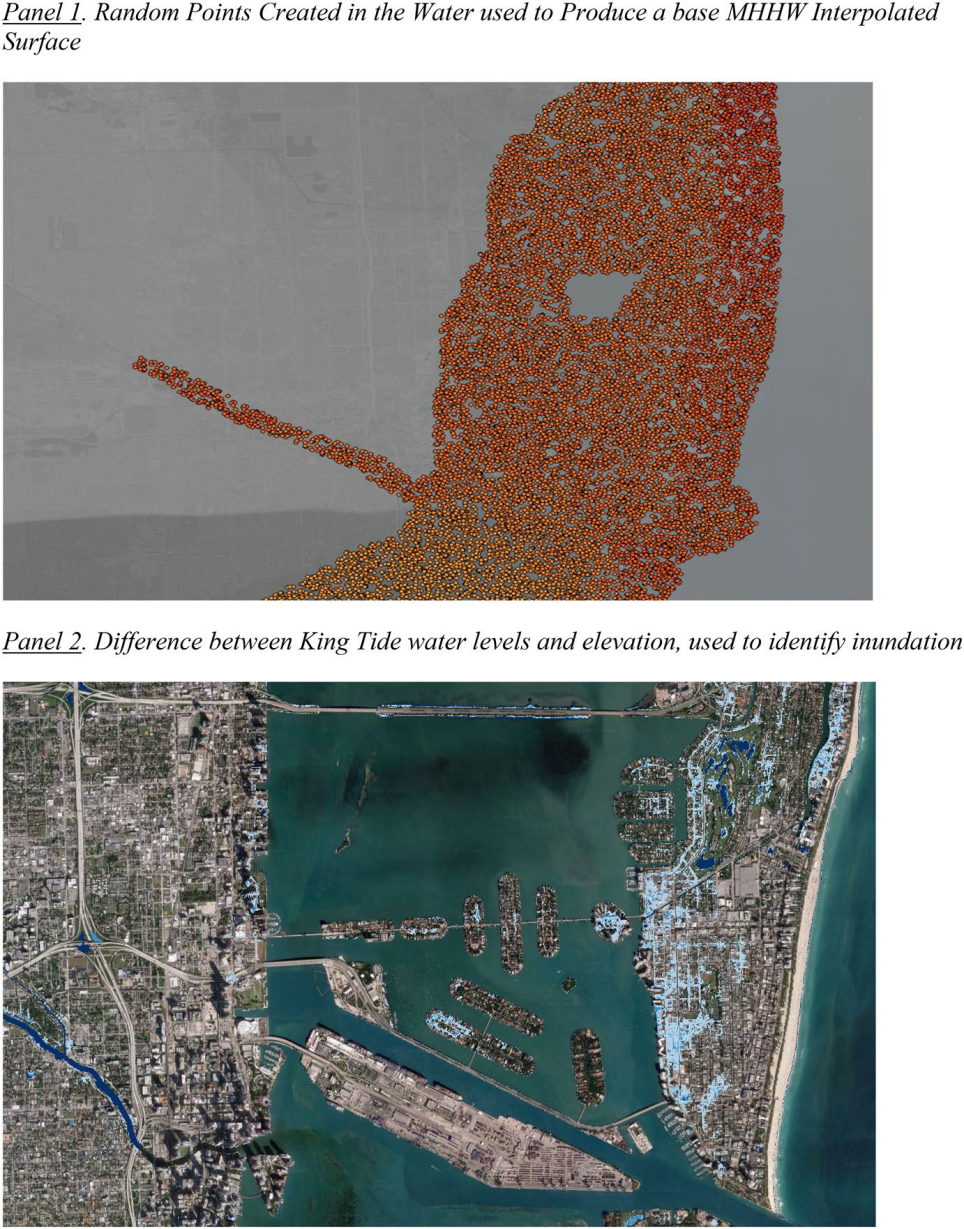
Development of tidal flooding variable

In comparison to storm surges, repeated tidal flooding risk is more predictable and offers a level of certainty that is likely to be built directly into the housing market of any community that experiences such flooding. To determine the extent to which high tides are projected to cause repeated inundation in the next few years, SLR projections, tidal variation patterns, and local sea-level elevations were combined to identify areas that will be below sea level when king tides (as they are commonly referred) cycle into any community and then forecasted for their arrival in future years. NOAA’s Vertical Datum Transformation tool was useful for determining the local mean sea level and local mean higher high water (MHHW) along the coast by creating random points in the water, sampling directly from NOAA’s VDatum tool, and interpolating a surface of MHHW (Fig. 2, Panel 1). The high-resolution interpolated surface was extrapolated inland and represented the highest average daily tide for any given geographic location.

Since the tool provides tidal datum values based on the last tidal epoch taken from tide readings between 1983 and 2001 (LMSL and MHHW), sea-level rise between the middle value of those years (1992) and today must be added to the elevations in order to determine current sea level (equal to plus 5 inches in 2017) and predicted sea level 15 years from now (equal to plus 11 inches in 2032) according to the United States Army Corps of Engineers High SLR curve for the Virginia Key Tide Gauge. Additionally, during certain times of the year, king tides raise high tide above these sea-level projections. To determine the extent to which tidal flooding can occur during king tide season, the seasonal variation of “higher high water” is added to the MHHW level. The king tide addition was calculated by comparing the top 20 highest tide days each year since 2000 against the average high tide of the year. That average difference, the mean of the top 20 tidal levels in each year relative to average annual high tide, was then added to the interpolated surface to determine elevations subject to repeated flooding levels in any given year.

For the Virginia Key tidal station in the South Florida region, since 2000 the average high tide for the 20 highest tide days was on average 10.5 inches higher than the average high tide of the year. This tidal variation was combined with the USACE sea-level curves and local MHHW levels in the NAVD88 projected datum to determine the projected height of average king tide sea levels, and inundation maps (Fig. 2, Panel 2) were created by differencing the DEM with this calculated king tide estimated surface. These inundation maps reflect an elevation of 21.5 inches above MHHW to show areas that in 2032 are projected to be subject to repeated tidal flooding of at least 10 days, which is also equivalent to areas that are projected to flood at least once a year in 2017.

### Calculating Property Flooding Statistics

**Fig. 3:**
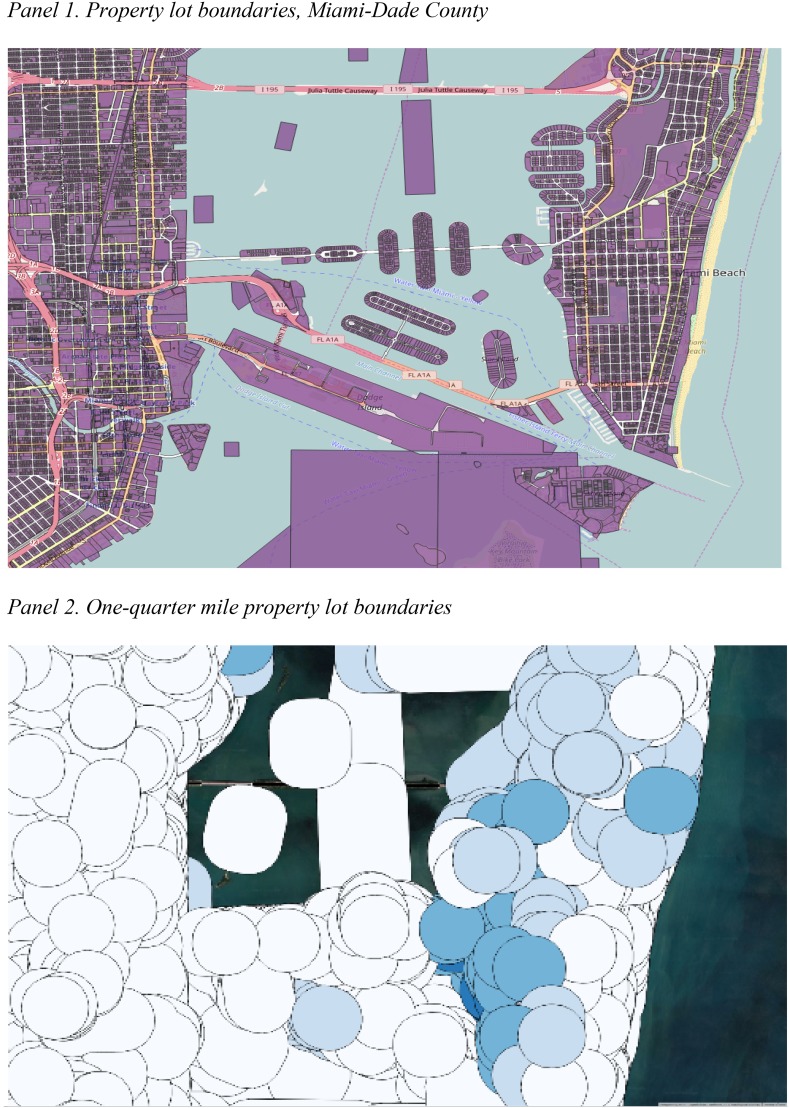
Property zones used in the creation of inundation statistics

Utilizing the tidal flooding and storm surge inundation-level raster grids at a 5-foot resolution in Miami-Dade, the proportion of property flooding indicators was created for tidal flooding inundation, Category 1 (C1) hurricane inundation, and Category 3 (C3) hurricane inundation from the time of the study. All three layers were created for 2017, 2022, 2027, and 2032; however, the only future projection used in the final regressions was the 2032 tidal flooding layer. Overlaying these high-resolution inundation raster grids with property lot boundary files (Fig. 3, Panel 1) allowed for the calculation of zonal statistics for each property lot. Specifically, the proportion of the property lot forecasted to be inundated under the tidal and hurricane scenarios was calculated based on the wet/dry cells within the property lot boundaries. In addition, the area of nearby roads inundated under each of the flooding scenarios was also calculated. This required a two-step process in which the inundation layers (wet/dry raster cells) were clipped by a GIS road file to produce a high-resolution raster grid that included only wet locations on roads surfaces. Each property’s local road inundation level was then calculated by producing the zonal statistics for the proportion of all road surface within a one quarter mile radius of each property. The one quarter mile neighborhood catchment area was created by using a simple polygon buffer boundary that extended the property lot boundaries by one quarter mile in each direction (Fig. 3, Panel 2).

**Fig. 4:**
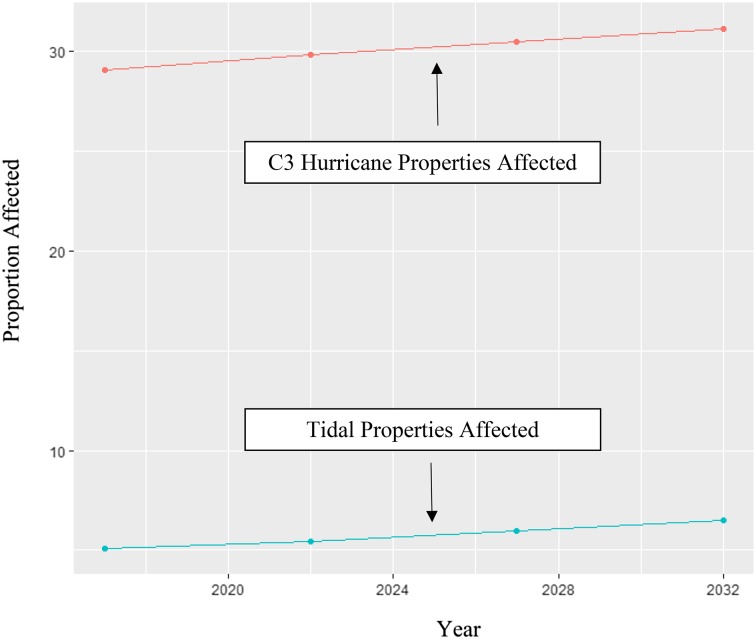
Proportion of lots affected by tidal flooding and C3 hurricane

The proportion of each property lot flooded was calculated for all three risk types and four time periods, 2017, 2022, 2027, and 2032. The trends associated with the proportion of properties affected by any level of a C3 hurricane and tidal flooding across these years are presented in Fig. 4. These trends show how a Category 3 hurricane could impact up to 29% of properties today but could grow to 31% in 15 years even before accounting for the additional velocity of the storm surge due to a decreased difference between surge height and land elevation (Fig. 4). The proportion of properties affected by tidal flooding is small, starting around 5% but increasing to 6.5% in 2032. To account for properties that naturally extend into water, any proportion of a property lot that was flooded under normal high tide scenarios (MHHW + SLR to 2017) was removed from the property lot inundation proportion calculation. These trends indicate that flooding from a C3 storm could be a significant concern for a nearly one out of every three homes in the housing market in Miami-Dade County and for about one out of every 16 homes due to tidal flooding for properties proximate to the coast.

### Estimating Financial Impact of Flooding Risk

After determining the flooding risks to each property lot, the inundation calculations were then combined with the larger property-level database that included information such as lot acreage, bedroom count, and total square feet of living area. According to an analysis of flooding events and media reports in Miami-Dade by Wdowinski et al. (2016) and backed up with empirical observations of MHHW levels from the NOAA tide gauge station site for Virginia Key, tidal flooding increased from just two major events between 1998 and 2005 to 16 major events between 2006 and 2013. The 2005 date should not be thought of as a definitively hard date for pre- and post-flooding occurrences, but it does identify two potential analytic time-frames for analyzing real-estate value growth. The year 2005 then serves as a baseline year to identify high value areas before tidal flooding became a more common occurrence in observation and in terms of their coverage in the local media.

**Fig. 5:**
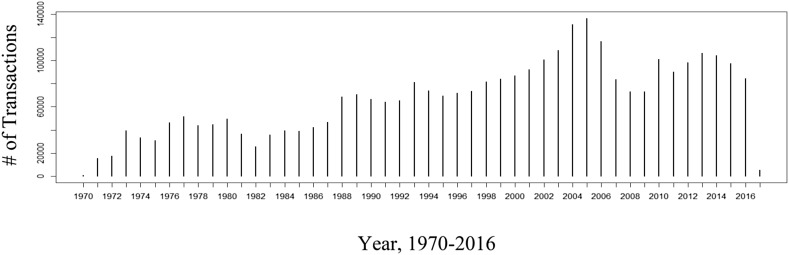
Number of real-estate transactions by year in Miami-Dade County

To measure the changes in values, a history of real-estate transactions was required. Fortunately, Miami-Dade County retains extensive real-estate transaction history of approximately 3.2 million transactions starting around 1970. Figure 5 represents the temporal trend in real-estate transactions from 1970 to 2016. From Fig. 5, one can clearly make out some of the macroeconomic trends surrounding well-known recessions and housing bubbles. These fluctuations were controlled in all models, so that any results would not be the artifact of larger temporal trends, through the use of yearly controls and appropriate polynomials. After excluding outlier cases, a price per living square foot metric provided a way to determine the overall value of an area.

**Fig. 6:**
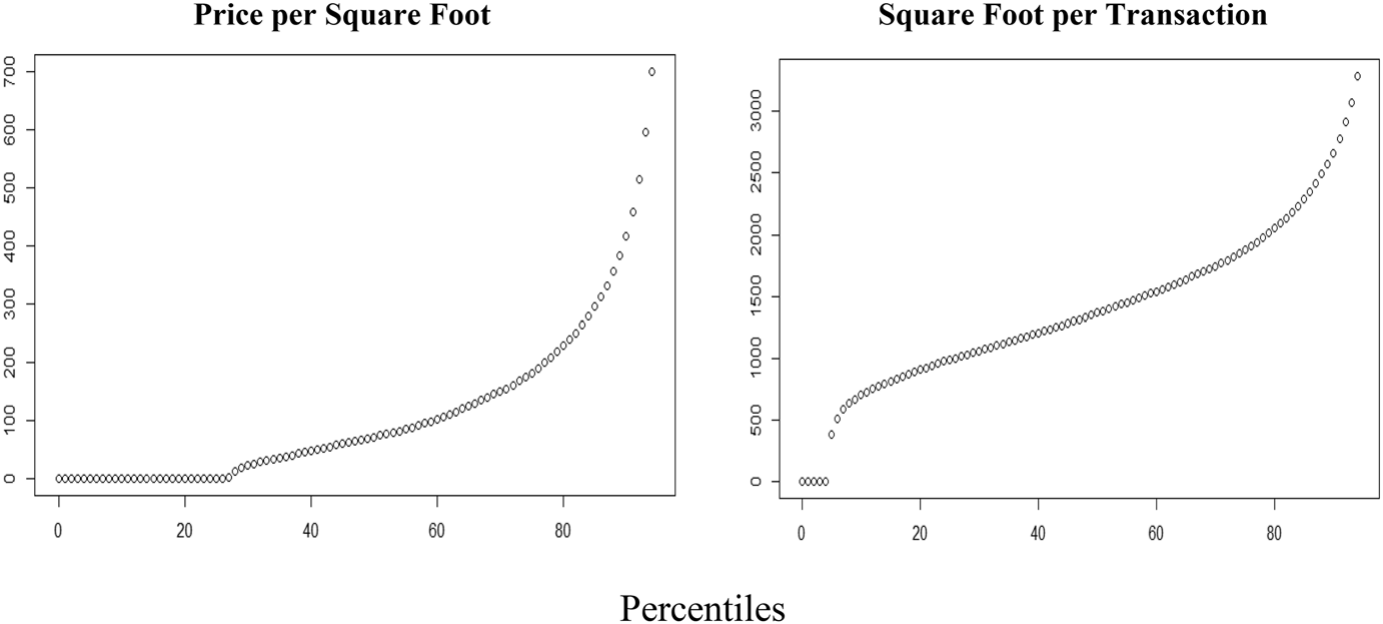
Transaction amount for each property sale showing many non-market value transactions. High tail of distribution is excluded from graph

Another consideration included the fact that many transactions were sold less than would be considered reasonable for property in the area. Figure 6 reports that there was a wide range of property lot sales values during this time period since some of the transactions reflect ownership changes within organizations, incomplete information, inflation, and the distribution of property values within Miami-Dade. About 27% of the transactions were dropped from consideration in the analysis because they reported prices were less than $1000 and were deemed to be not normal transactions between individuals or organizations purchasing properties from other entities at fair market value.

**Fig. 7:**
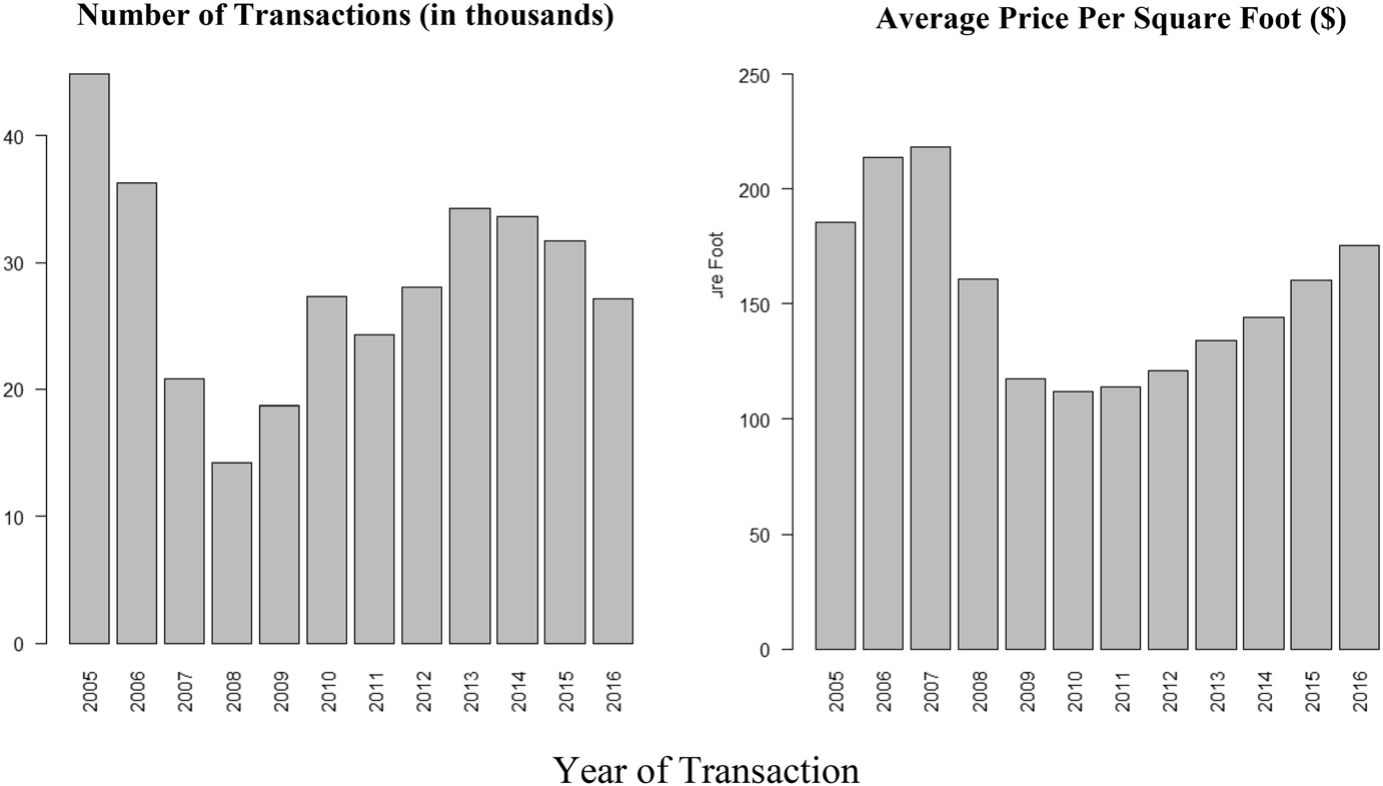
Number of transactions and average price per square foot from 2005

The square footage of parcels follows a more even distribution with only some lots having zero square feet of living area. These lots are mostly undeveloped government-owned land and thus were also excluded. After excluding these outlier cases, a price per living square foot metric provided a way to determine the overall value of an area. Figure 7 presents the distribution of the sample of interest to this analysis and in reference to the number of transactions and Average Price per Square Foot for real-estate transactions in Miami-Dade *after* 2005.

### Property Value

#### Dependent Variable

**Fig. 8:**
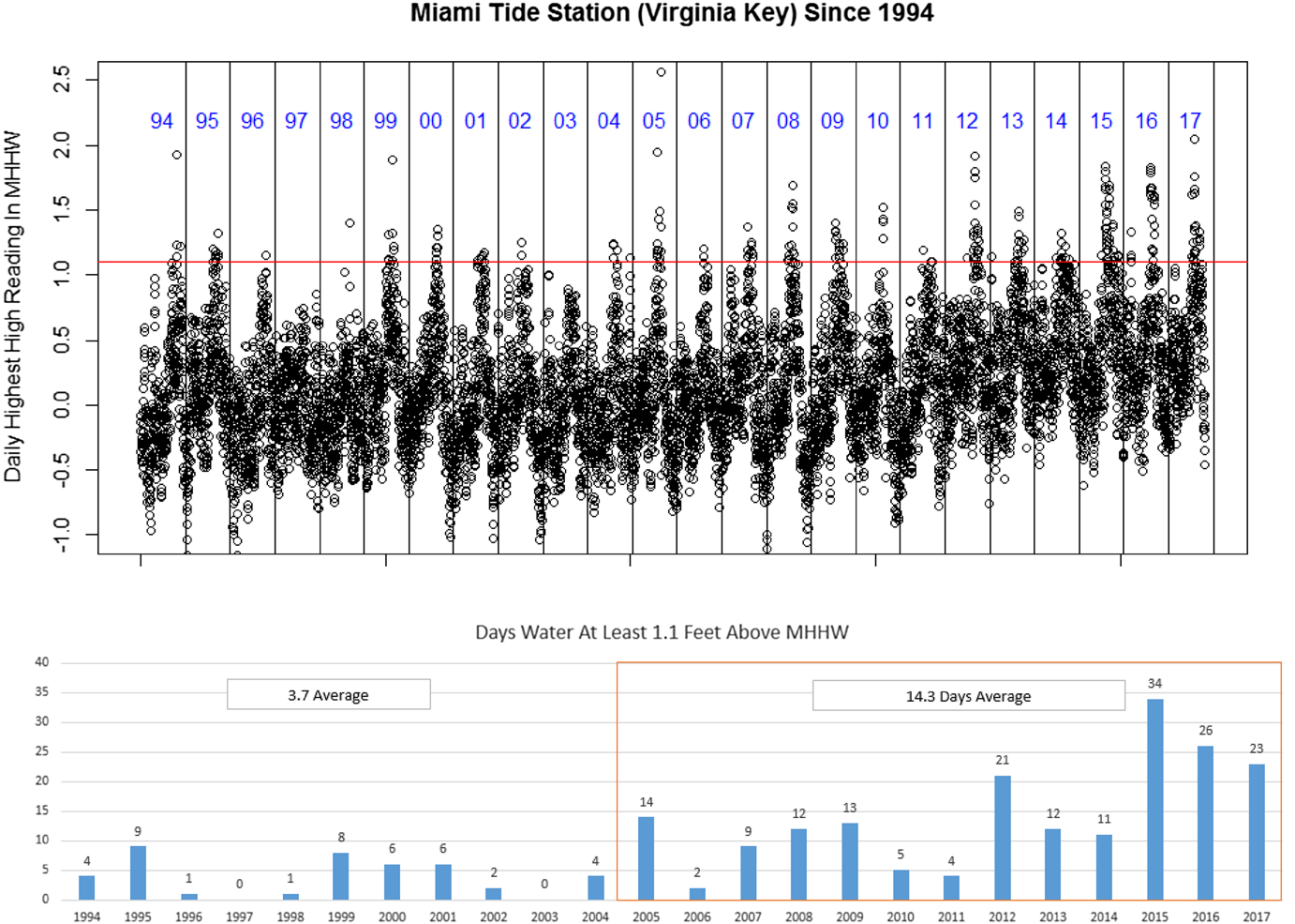
Observed MHHW Level and number of days in which water level rose above nuisance flooding level (1.1 foot about MHHW) at the Virginia Key Station from 1994 to 2017

The dependent variable of interest in this analysis was the price per square foot of all property sales between the years of 2005 and 2016 that were deemed to be real property transactions. Each property could have multiple sales over the 12-year period, but only one sale per year was included in the analysis. In the event that multiple sales occurred in a single year, only the most recent sale for that year was included in the analysis. Figure 8 highlights the observed increase in flooding events in the Miami-Dade area.

#### Spatio-Temporal Trends and Covariates

**Fig. 9:**
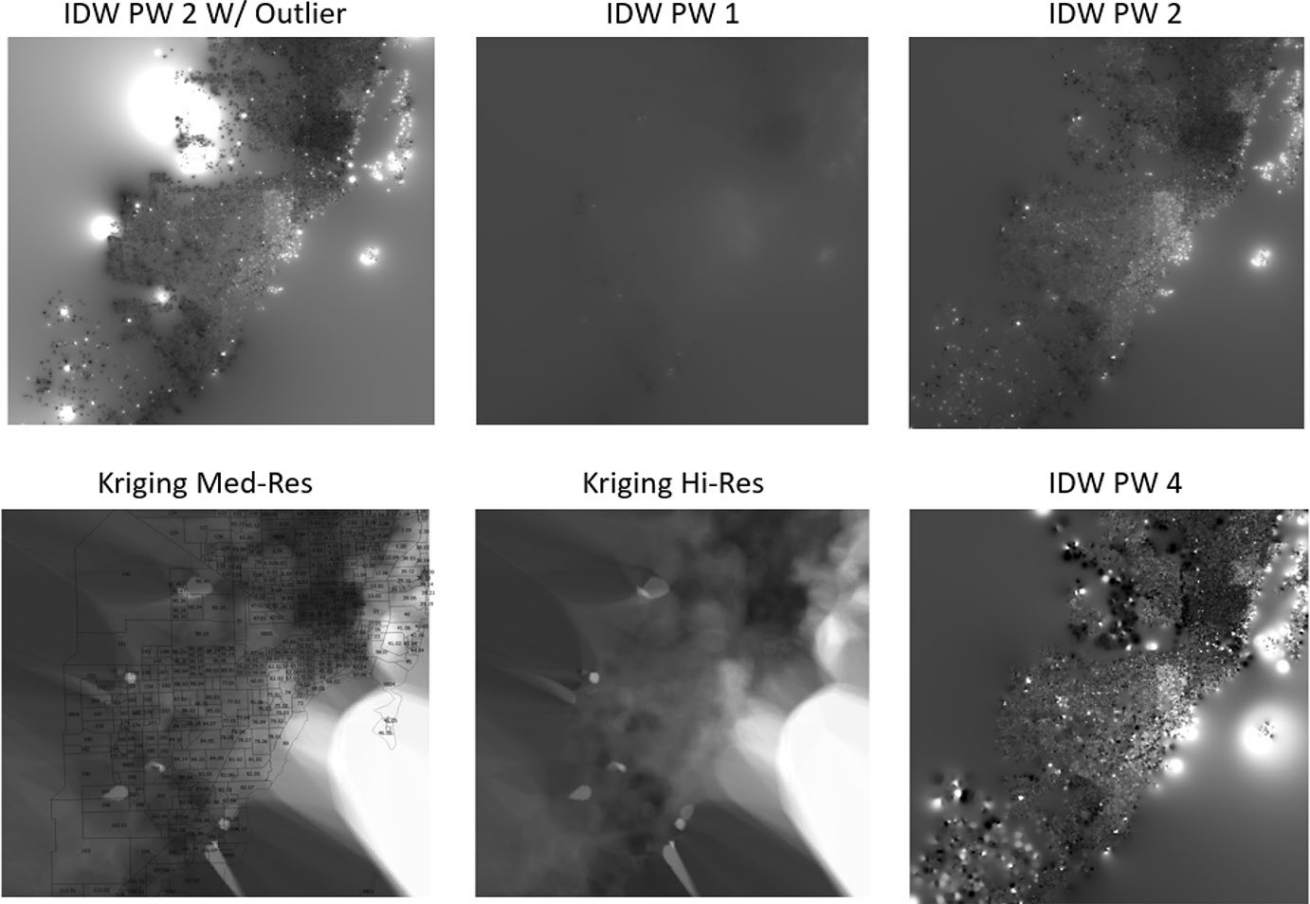
Price per square foot interpolation maps based on property sales from 2005

In order to account for the baseline spatial variation identified at the property level, property value surfaces were built for comparison using different interpolation techniques. These were created by first calculating the average price per square foot of living area for each property sold in 2005. After limiting the analysis to valid, non-outlier lots, there were ~ 41,000 data points to use for creating each property value surface. The results of the property value surface estimates are presented in Fig. 9 and highlight the strengths and weaknesses of the different methods. Ultimately, we chose to use the Inverse Distance Weighted (IDW) 2nd order polynomial interpolation surface as being the most representative baseline of the actual underlying property value variation in the county in the baseline year of 2005. Ultimately, the result of this process allows for the estimation of baseline property values for the neighborhood that will further vary based on information associated with each property concerning the square footage, number of bedrooms, etc.

Although generally moving with the macroeconomic trends, housing values in some census tracts grew at a faster rate than overall trends, while values in other tracts declined or grew at a slower rate relative to overall trends. These uneven growth trends resulted in a distribution of average housing values by tract that deviate from overall value growth trends, with some tracts having grown more than $50/square foot versus overall trends, and others declining more than $50/square foot versus overall trends.

#### Fixed Effect Linear Models

Explanatory models had to test for, and account for, uneven property value trends by census tract. Since land value did not grow or decline uniformly across all areas, as some regions of the county became more valuable while others declined in value, this uneven change was captured in the modeling process by allowing varying slopes for each tract using an interaction between the years since 2005 and the census tract of each property. These varying slopes were estimated for each census tract using both fixed effects (no pooling) and random effects (partial pooling), to test parameter robustness versus pooling assumptions. The characteristics and zoning of each individual lot is also correlated with the sale price of the lot. Large lots with more land sell for higher prices and newer buildings are generally worth more, while a large number of units generally devalue the property, and residential lots are evaluated differently versus commercial and industrial lots. The contribution of these components to property value was modeled by including lot acres, number of living units, zoning codes, and age of the property. Property age was accounted for by standardizing the values with mean centered at 0 and values 1 standard deviation away as 1 and − 1, 2 deviations away as 2 and − 2, etc., then regressing on 1st and 2nd order polynomials of these standardized values. This accounted for the original non-linear relationship between property age and home value.

#### Data Processing Summary

**Fig. 10:**
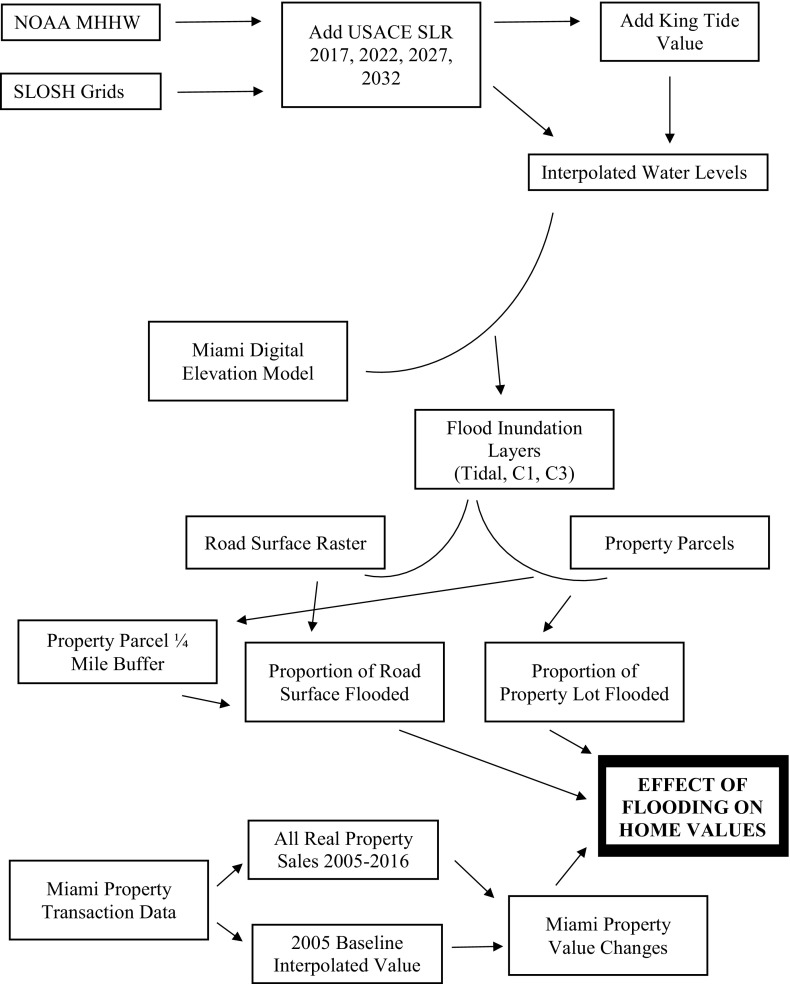
Data management flow to produce flooding layers

In summary, Fig. 10 documents the overall data processing summary followed to produce the flooding layers and ultimately to estimate the relationship between flooding inundation from tidal and hurricane sources and property values relative to their 2005 base. From the top of the figure, we began with data from NOAA pertaining to the MHHW and SLOSH inundation levels. For tidal flooding, the USACE SLR estimates were added to the repeated tidal flooding variation determined by analyzing the top 20 readings in each year since 2000. Those data were interpolated to produce a “water level” surface, which was differenced against the Miami-Dade 5-foot elevation model. The resulting inundation layer was then converted into a binary format for wet/dry land areas, and this was also used to create a wet/dry road surface layer. Using a zonal statistics operation, the proportion of property inundated in 2017 and 2032 was calculated for both tidal and hurricane sources and the method was repeated for wet/dry roads within a ¼ mile buffer of the property. These flooding inundation indicators were then used to explain the price per square foot in all real-estate transactions in Miami-Dade between 2005 and 2016 controlling for the baseline 2005 property values. The primary variables included in the full regression models are presented in Table 1.

**Table 1:** Descriptive statistics

Variable	Mean (%)
Dependent variable	
Price per Square Foot	$157.03
Controls	
Years since 2005 (YR05)	5.43
Acres	0.22
Bedrooms	3.51
Year built	1976
Living units	1.37
Near coast	20%
Near golf course	49%
Near park	91%
Average flooding inundation of lot, for all properties	
Lot tidal flooding 2032	< 1%
Road tidal flooding 2032	1%
Lot C1 flooding 2017	3%
Lot C3 flooding 2017	29%

## Results

While controlling for the spatial–temporal variations and additional lot-specific predictors of property values, it was possible to estimate the independent effects on property values from tidal flooding and hurricane storm surge risks. The parameter of interest, the interaction between tidal flood risk and years since 2005, is consistently negative and statistically significant in various modeling configurations. The models explaining the most variance in the property value indicator (adjusted *r*^2^ 51.2%) are presented in Tables 2 and 3. The first thing we want to draw attention to is that before interacting with years there are consistently positive estimates for the baseline tidal flooding risk coefficients. This is due to the fact that high-risk properties are also in the most desirable coastal areas, and therefore in 2005 these properties commanded a higher price than other nearby properties. In order to understand how this desirability has changed in relation to changing lot and/or road flooding risk, the interaction of the tidal flooding variables and year since 2005 was included in the model (*YR05:KT32* and *YR05:Road KT32*, respectively). In the first model, the estimated effect on property values of the interaction between lot flooding risk and years since 2005 is − $3.757 per square foot of living area, per year since 2005 for a lot that is forecasted to be completely flooded during king tide season in 2032 (Table 1, Model 1). The interpretation of this result indicates that there is a discount due to tidal flooding risk that is growing over time and is related to the amount of exposure of a lot. This is evidence there is a market response to current flooding and the potential for increased flooding on these properties in the near future, and this response could be related to the increased media coverage (Wdowinski et al. 2016) or to the observable experience of rising seas and increasing flooding in the locales affected by tidal flooding.

**Table 2:** Regression estimates predicting relative property value change by indicators of future property flooding

	Dependent variable		
	Price per square foot of living area		
	Model 1	Model 2	Model 3
Control variables			
2005 area property value	60.58*** (0.30)	60.58*** (0.30)	60.58*** (0.30)
(Years since 2005)^2^	− 60.29*** (1.66)	− 60.27*** (1.66)	− 60.27*** (1.66)
(Years since 2005)^3^	12.39*** (0.15)	12.39*** (0.15)	12.39*** (0.15)
(Years since 2005)^4^	− 1.03*** (0.01)	− 1.03*** (0.01)	− 1.03*** (0.01)
(Years since 2005)^5^	0.03*** (0.01)	0.03*** (0.01)	0.03*** (0.00)
Years since 2005 (YR05)	118.49*** (13.19)	118.53*** (13.19)	118.50*** (13.19)
Acres	0.91*** (0.11)	0.91*** (0.11)	0.91*** (0.11)
Bedrooms	− 0.12*** (0.02)	− 0.12*** (0.02)	− 0.12*** (0.02)
Year built standardized	2.26*** (0.23)	2.27*** (0.23)	2.28*** (0.23)
Year built standardized^2^	5.35*** (0.20)	5.36*** (0.20)	5.36*** (0.20)
Living units	− 0.21*** (0.02)	− 0.21*** (0.02)	− 0.21*** (0.02)
Near coast	58.71*** (1.48)	59.25*** (1.47)	58.79*** (1.48)
Near golf course	26.19*** (1.06)	26.27*** (1.06)	26.18*** (1.06)
Near park	− 3.95*** (1.23)	− 4.04*** (1.23)	− 3.93*** (1.23)
Flooding indicators			
Lot tidal flooding 2032 (KT32)	51.95*** (8.35)	31.22*** (3.59)	52.10*** (8.354)
YR05:KT32	− 3.75*** **(1.10)**	**–**	− 3.08*** **(1.113)**
YR05:Road KT32	**–**	− 4.06*** **(0.86)**	− 3.71*** **(0.875)**
Changes in amentity value controls			
YR05: near coast	− 4.356*** (0.413)	− 4.28*** (0.41)	− 4.23*** (0.41)
YR05: near golf course	− 3.395*** (0.251)	− 3.42*** (0.25)	− 3.41*** (0.25)
YR05: near park	0.031 (0.282)	0.02 (0.28)	0.01 (0.28)
Observations	341,354	341,354	341,354
*R* ^2^	0.51	0.51	0.51
Adjusted *R*^2^	0.51	0.51	0.51
Residual std. error	80.76	80.76	80.76
*F* statistic	334.86***	334.88***	334.58***

Bold indicates the impact of current and future flooding levels on relative property value appreciation

Not shown are fixed effect intercepts for property zone type and the interaction between census tract and years since 2005, and additional linear distance interactions with the coast, golf course, and park variables

*p < 0.1; **p < 0.05; ***p < 0.01

**Table 3:** Regression estimates predicting relative property value change by indicators of future property flooding and current hurricane flooding

	Dependent variable		
	Price per square foot of living area		
	Model 1	Model 2	Model 3
Control variables			
2005 area property value	60.58*** (0.30)	60.54*** (0.30)	60.37*** (0.30)
(Years since 2005)^2^	− 60.29*** (1.66)	− 60.35*** (1.66)	− 60.57*** (1.66)
(Years since 2005)^3^	12.39*** (0.15)	12.40*** (0.15)	12.43*** (0.15)
(Years since 2005)^4^	− 1.03*** (0.01)	− 1.03*** (0.01)	− 1.04*** (0.01)
(Years since 2005)^5^	0.03*** (0.01)	0.03*** (0.01)	0.03*** (0.01)
Years since 2005 (YR05)	118.49*** (13.19)	118.96*** (13.19)	119.12*** (13.18)
Acres	0.91*** (0.11)	0.92*** (0.11)	0.91*** (0.11)
Bedrooms	− 0.12*** (0.05)	− 0.12*** (0.05)	− 0.12*** (0.02)
Year built standardized	2.26*** (0.23)	2.24*** (0.23)	3.02*** (0.24)
Year built standardized^2^	5.35*** (0.20)	5.34*** (0.20)	5.45*** (0.20)
Living units	− 0.21*** (0.02)	− 0.21*** (0.02)	− 0.21*** (0.02)
Near coast	58.71*** (1.48)	56.94*** (1.53)	66.61*** (1.52)
Near golf course	26.19*** (1.06)	26.24*** (1.06)	23.40*** (1.06)
Near park	− 3.95*** (1.22)	− 3.84*** (1.23)	− 2.05* (1.23)
Flooding indicators			
Lot tidal flooding 2032 (KT32)	51.95*** (8.35)	41.89*** (8.68)	64.73*** (8.37)
Lot C1 flooding 2017 (C117)	–	9.48*** (2.17)	–
Lot C3 flooding 2017 (C317)	–	–	− 16.01** (0.76)
YR05:KT32	− 3.75*** **(1.10)**	− 2.37** **(1.13)**	− 5.36*** **(1.10)**
YR05:C117	**–**	− 1.62*** **(0.33)**	**–**
YR05:C317	**–**	**–**	2.52*** **(0.15)**
Changes in amenity value controls			
YR05: near coast	− 4.35*** (0.41)	− 4.09*** (0.41)	− 5.55*** (0.41)
YR05: near golf course	− 3.39*** (0.25)	− 3.39*** (0.25)	− 3.06*** (0.25)
YR05: near park	0.03 (0.28)	0.02 (0.28)	− 0.16 (0.28)
Observations	341,354	341,354	341,354
*R* ^2^	0.51	0.51	0.51
Adjusted *R*^2^	0.51	0.51	0.51
Residual std. error	80.76 (df = 340,281)	80.76 (df = 340,279)	80.71 (df = 340,279)
*F* statistic	334.86*** (df = 1072; 340,281)	334.28*** (df = 1074; 340,279)	335.12*** (df = 1074; 340,279)

Bold indicates the impact of current and future flooding levels on relative property value appreciation

Not shown are fixed effect intercepts for property zone type and the interaction between census tract and years since 2005, and additional linear distance interactions with the coast, golf course, and park variables

*p < 0.1; **p < 0.05; ***p < 0.01

The average property predicted to be affected by repeated tidal flooding in 2032 has 2633 square feet of living area, and the proportion of flooding by king tides in 2032 in the average affected lot is predicted to be 12.9%; thus, given the regression estimate, the average affected lot has lost $1276.09 in value each year since 2005. Extrapolated to the end of the analysis period, on average each affected lot has lost $14,037.00 of the on-average $722,000 in total property value due to the risk of tidal flooding.

Table 2, Model 2 replaces the interaction between years since 2005 and lot flooding with a similar interaction between year and the proportion of nearby roads that are predicted to be flooding. The statistically significant estimate of − $4.061 indicates a yearly loss in value associated with proximity to roads that are currently flooding or are likely to repeatedly flood in the near future. Model 3 simultaneously estimates the independent effects of nearby road repeated tidal flooding inundation and predicted lot tidal flooding inundation by interacting each with the years since 2005. Here we see negative and statistically significant yearly effects for both predicted tidal flooding on lots − $3.078 and in predicted tidal flooding in nearby roads − $3.712.

Lastly, to compare the different risk types against one another, two additional regression models adding current (2017) risk from a Category 1 hurricane or risk from a Category 3 hurricane were also estimated (Table 3, Models 1–3). The results of the regressions show the degree of similarity between risk from a Category 1 hurricane at the time of the study and forecasted future tidal flooding (Table 2, Model 2); the results also show that there is additional property value being lost to areas safe from repeated tidal flooding risk in 15 years, but at low enough elevations near the coast to be at risk from a C1. Properties at risk of tidal flooding are generally also at risk of inundation from hurricane storm surge, as evidenced by the year-over-year loss per square foot of property value of $2.37 due to forecasted tidal flooding (YR05:KT32) and an additional $1.62 due to potential C1 hurricane storm surge (YR05:C117). Given that the two can be independent of one another but many lots are at risk of both, a property at risk of both future tidal flooding and current C1 storm surge would, on average, have an associated $3.99 loss per square foot for each year since 2005. The properties residing in the zones between complete inundation under a C3 and complete inundation under tidal flooding in 2032 are actually increasing in value at $2.53 per square foot. These properties are generally at high enough elevations that it is possible buyers perceive them to be safe from flooding. This could be the same correlation between higher elevation and price appreciation identified by Keenan et al. (2018).

Our results most directly complement the recent research by Keenan et al. (2018) in the Miami-Dade area, which showed that not only are property values being affected by elevation and the perceived protections associated with it, but population redistribution and its associated investment patterns in real estate are shifting as well. The results pinpoint the source of the decrease value at lower elevation, either flooding within lots or flooding on the roads nearby to lots. Both our work and the work of Keenan et al. (2018) are consistent with those researching sea-level rise and its future impact on real-estate losses in coastal North Carolina (Bin et al. 2011) and Tampa, FL (Fu et al. 2016), but go further by providing evidence that sea-level rise is affecting home values today versus in the decades ahead. In addition, our research further adds to this line of literature by more precisely measuring current flooding inundation levels for each property and looks beyond property lot flooding by taking into account the inundation levels of nearby roads.

## Discussion and Conclusions

We find empirical support for significant and negative impacts, in property value appreciation due to the increasing risks of tidal flooding. This is likely to be linked to both observable increases in flooding events for locales within the Miami-Dade region as well as the documented increases in media coverage of these events. The negative effect of predicted C1 lot inundation provides evidence that low-lying areas outside the zones expected to be experience repeated tidal flooding are also becoming less valuable. This could be due to the fact that buyers are also worried about SLR effect on storm surges or more generally worried about the risks associated with coastal property in low-lying areas. In both flooding scenarios, the increased incidence and awareness of flooding seem to serve as mechanisms to decrease or restrict property value appreciation over the study period.

Although the specific flood inundation risk measures presented in this research were not available to the public, lot purchasers are becoming aware of the risk through other sources. The other sources of information are the mechanism through which flooding risk has suppressed house values. These other sources might be photos and accounts of flooding occurring on the streets near the properties, or photos and accounts of flooding within the property boundaries. This evidence could also exist because many of the areas projected to experience regular tidal flooding are already flooding occasionally, so prospective buyers could also have first-hand accounts. To be more specific, the tidal flooding layer used produced only inundation for repeated flooding events, or those that would flood at least 10 times a year in 2032, but many of those properties likely already flood once or twice a year today. In addition, some of these areas may flood during heavy rain events due to poor runoff in the gravity-based drainage systems. More generally, the mechanism may be through evidence of past flooding, but could also be due to anecdotal buyer awareness of low-lying areas within or near the property lots. Additionally, prospective buyers may have commissioned engineering firms or other consulting agencies to determine the flooding risk of lots under consideration.

Irrespective of the mechanism through which buyers are becoming aware of the risk, since 2005 the estimated total amount of lost real-estate value due solely to future near-term tidal flooding of property lots in this analysis totals to − *$115,684,000*. The total loss figure grows about four times larger once the lost value due to future near-term road flooding is included, an additional − *$349,906,000*. This combined loss of − *$465,554,000* in value is relatively small compared to the hundreds of billions of total real-estate value in Miami-Dade; however, without significant intervention, this value is likely to increase. The results also indicate that our current understanding of the impact of flooding on real-estate prices is likely undervaluing the potential losses or projecting the losses to occur too far in the future. Here we show that even small proportions of lot inundation and road inundation from tidal sources are likely to negatively impact home values. The nearby neighborhood road flooding is especially problematic, as it means properties that are not forecasted to be permanently lost (underwater) to rising seas can still lose value. This is a significant finding that indicates the economic impact of SLR, minus adaptation costs, could be much larger than currently estimated.

This research provides evidence that real-estate values are being affected by the risks associated with sea-level rise, so property owners, potential buyers, and governments will have to take note of the current trends. Property owners should by informed of property characteristics concerning their elevation and flood risk in order to make informed decisions about employing adaptation strategies individually or pushing for adaptation strategies at a governmental level. At the local government level, concerns about what this impact might do to the local tax base are of import. While the amounts of lost value to date are small relatively to the total value in Miami-Dade, if loss trends continue or accelerate it could begin to put a strain on certain municipalities. This is especially true for smaller municipalities such as North Bay Village, in Miami-Dade, or even in larger municipalities outside Miami-Dade that are already experiencing high levels of flooding like Norfolk, VA, or Charleston, South Carolina. Generally, larger cities such as the city of Miami will be able to function despite the loss of areas directly impacted by SLR; it is those without larger portions of their population living away from the coast that are likely to struggle and need a more immediate response to the issue of rising seas and their impact on home owners. In order to preserve property values and the historical continuity of neighborhoods in coastal communities, individuals and government officials should look for strategies to reduce the risk of tidal flooding through reasonable measures of adaptation on both small and large scales. Since there are numerous stakeholders impacted by this impending and worsening trend, it is important that individuals, communities, and governments work together to tackle this issue as soon as possible.
